# Deciphering Circadian Rhythm–Related Molecular Subtypes in Breast Cancer and Establishing a Prognostic Prediction Model

**DOI:** 10.1155/bmri/9664238

**Published:** 2025-09-09

**Authors:** Yanzhen Lu, Yunfei Yang, Dan Yu, Yuxi Tan, Gang Feng, Liquan Ouyang, Lulu Tan, Yuyan Tan

**Affiliations:** ^1^ Department of Thyroid and Breast Surgery, The First College of Clinical Medical Science, China Three Gorges University & Yichang Central People’s Hospital, Yichang, Hubei, China, ctgu.edu.cn; ^2^ Central Laboratory, The First College of Clinical Medical Science, China Three Gorges University & Yichang Central People’s Hospital, Yichang, Hubei, China, ctgu.edu.cn; ^3^ China Three Gorges University, Yichang, Hubei, China, ctgu.edu.cn

**Keywords:** breast cancer, circadian rhythm, immune checkpoint, immunotherapy, tumor microenvironment

## Abstract

**Background:** Recent evidence suggests that alterations in circadian rhythm genes may lead to circadian rhythm disruption (CRD), which is a key mechanism in the progression of breast cancer. Therefore, investigating the role of circadian rhythm genes in the prognosis of breast cancer holds significant clinical value.

**Materials and Methods:** We utilized expression profile data from the Gene Expression Omnibus (GEO) database to identify cancer features closely associated with CRD in breast cancer. Then, we analyzed publicly available datasets (including GEO, TCGA, and METABRIC) to identify alterations in core circadian genes significantly associated with patient survival across breast cancer and constructed a circadian‐related gene signature (CGS) based on these prognostic cancer features.

**Results:** Circadian rhythm–related genes (CRGs) were selected to construct a risk gene signature associated with individual prognosis, which was validated in six independent cohorts and demonstrated good predictive ability. We identified three circadian rhythm–associated subtypes with distinct prognoses, which exhibited significant differences in immune checkpoint molecules, drug sensitivity, and molecular features. Additionally, the gene signature and clinicopathologic features were integrated to develop a risk model with enhanced predictive accuracy. To validate the functional role of signature genes, BMAL1 knockdown in SKBR3 cells disrupted circadian rhythms, with qPCR confirming altered risk gene expression. We found that the nomogram exhibited superior discriminative ability compared to the traditional breast cancer staging system.

**Conclusion:** We developed a nomogram that can accurately predict the prognosis of breast cancer, and conclude that the expression of CRGs is crucial in breast cancer treatment decisions.

## 1. Introduction

Breast cancer is the most common cancer among women and a leading cause of death worldwide [1]. Due to considerable genetic and histopathologic heterogeneity, standard chemotherapy/targeted therapies may not be effective for all patients [2]. Therefore, exploring precision medicine and effective prognostic prediction methods is a key focus in breast cancer research. With the advancement of next‐generation sequencing technologies, biochemical and genomic studies on circadian rhythm functions have emerged as a hot topic [3–5]. Circadian rhythm genes are the core regulatory mechanisms of the body’s biological clock. Research has shown that mutations in circadian rhythm genes are closely associated with the development of breast cancer [6–8], and different circadian rhythm pattern subtypes exhibit distinct immune landscapes [9, 10]. Some subtypes are associated with high immune cell infiltration and antitumor microenvironment responses, while others are linked to immune suppression and poor prognosis [11].

In this study, we analyzed mutations and dysregulation of circadian rhythm genes in breast cancer samples from The Cancer Genome Atlas (TCGA) database, identifying three circadian rhythm–related subtypes in breast cancer. Furthermore, we developed a circadian rhythm gene‐based scoring system to predict breast cancer prognosis. Subsequently, by integrating genetic signatures and clinicopathological characteristics, we established a risk model with high prognostic accuracy and predictive capability, which demonstrated superior prognostic accuracy compared to traditional breast cancer staging systems. Overall, our results further elucidate the critical role of circadian rhythm genes in personalized treatment and prognostic evaluation in patients with breast cancer.

## 2. Materials and Methods

### 2.1. Data Collection and Preprocessing

The gene expression profiles of BRCA tissues from four publicly available datasets (GSE42568, GSE61304, GSE2034, and GSE21653) were retrieved from the NCBI Gene Expression Omnibus (GEO) repository (https://www.ncbi.nlm.nih.gov/geo/). TCGA‐BRCA normalized data and clinical information were downloaded from the UCSC Xena website (https://xenabrowser.net). All gene expression profiles were normalized by R software. All samples in each cohort were standardized to remove batch effects.

### 2.2. Unsupervised Clustering Analysis of Circadian Rhythm–Related Genes

Prognosis‐related CRGs were filtered out using univariate Cox regression analysis with the R packages “survival” and “survminer.” Breast cancer patients were divided into three CRG clusters based on the prognosis‐related CRGs by consensus unsupervised clustering analysis with the R package “Consensus ClusterPlus.” The univariate Cox regression analysis was performed to identify the prognosis‐related differential expression genes (DEGs). Based on the prognosis‐related DEGs, breast cancer patients were divided into three gene subtypes by consensus unsupervised clustering analysis. The criteria were as follows: (1) balanced sample distribution across groups, (2) smooth incremental progression of cumulative distribution function (CDF) curves with plateau stability, and (3) intracluster linkage strength > 0.9 while intercluster linkage strength < 0.1.

### 2.3. Identification of Differentially Expressed Genes

All raw data were normalized and standardized by using the R software package. Gene differential expression analysis was conducted through the “limma” packages in the Bioconductor package (available online: http://www.bioconductor.org/). FDR (false discovery rate) *q* − value > 0.05 and log2 (|fold change (FC)|) > 1.5 were set as cutoff standards and considered to indicate statistical significance.

### 2.4. Gene Set Enrichment Analysis

The R package “GSVA” was used to perform single‐sample gene set enrichment analysis (ssGSEA), which was used to explore the enrichment of tumor‐related pathways and immune cell infiltration in the TCGA database. Tumor‐related datasets were obtained from hallmark gene sets in the MSigDB database. The characteristic gene set containing 28 immune cell types was downloaded from a recent publication.

### 2.5. Establishment and Validation of a Breast Cancer Prognostic Predictive Signature

Univariate Cox regression analysis was conducted to identify recurrence‐free survival (RFS) and overall survival (OS)–related cancer hallmarks.

### 2.6. Construction of a Nomogram for Breast Cancer Prognosis Prediction

To select circadian rhythm–related genes associated with prognosis, LASSO‐penalized Cox regression analysis was applied. The LASSO Cox regression model was employed to identify circadian rhythm highly correlated genes and to construct the prognostic CGS. For each patient, the coefficients of logistic regression were used to calculate the CGS score via the following formula: CGS score = ∑(coefficient × mRNA expression). Circadian rhythm score and relevant clinical parameters were used to construct a nomogram, using the survival and the “rms” package in R. The nomogram was constructed to estimate 1‐, 3‐, and 5‐year survival probabilities. The calibration curve and C index were used to evaluate the performance of the model.

### 2.7. Stromal and Immune Cell Infiltration

We used xCell to estimate the cellular composition of stromal cells and immune cells in tumor samples from the TCGA dataset. Immune and stromal cell scores for each sample were calculated. The CIBERSORTx online website was used to evaluate the infiltration of 22 immune cells in each sample. The absolute abundance of eight immune cell populations of tissue‐infiltrating immune cells was calculated by the R package “MCPcount.”

### 2.8. Drug Sensitivity Analysis

The GSCALite database was used for drug sensitivity analysis. The relationship between the expression levels of 12 target genes and drug sensitivity was calculated by Spearman correlation analysis.

### 2.9. Additional Bioinformatic and Statistical Analysis

Principal component analysis (PCA) was used to visualize the differences between different groups. The “GSVA” and “GSEA” packages were used to analyze pathway enrichment between the three subtypes. The “pRophetic” package was used to predict half‐maximal inhibitory concentration (IC_50_) values of BRCA therapeutics. Tumor immune dysfunction and exclusion (TIDE) [12] and exclusion algorithms were used to predict potential immune checkpoint blockade treatment response. All statistical analyses were performed using R Version 4.0.3. The Wilcoxon test was used for special variables (risk score, circadian rhythm–related genes, and circadian rhythm–related gene clusters) between the three groups. The chi‐square test was used for categorical variables between the three groups, and *p* < 0.05 was considered to be statistically significant.

### 2.10. SKBR3 Cell Culture and Treatment

The human SKBR3 breast cancer cell line, used for in vitro experiments, was purchased from Wuhan Pricella Biotechnology. Cells were cultured and maintained in complete medium containing Dulbecco’s modified Eagle’s medium (DMEM), high glucose, 10% fetal bovine serum, 100 U/mL penicillin, and 100 U/mL streptomycin. The SKBR3 cells were incubated at 37°C in a humidified incubator with 5% CO_2_, and the culture medium was replaced every other day. The BMAL1‐targeting shRNA sequence (5 ^′^‐GGGAAGCTCACAGTCAGATTG‐3 ^′^) was synthesized by GeneChem (Shanghai, China) and cloned into the GV493 lentiviral vector. Lentivirus was packaged in 293 T cells using a three‐plasmid system, with viral titers determined by qPCR (3.0 × 10^9^ TU/mL). SKBR3 cells were infected with lentivirus at an MOI of 40, and stable cell lines were selected with puromycin (2 *μ*g/mL) for 72 h.

### 2.11. Quantitative Real‐Time Polymerase Chain Reaction PCR (RT‐qPCR)

Total RNA was extracted from breast cancer cells (SKBR3) using TRIzol reagent (Invitrogen, Thermo Fisher Scientific, Waltham, Massachusetts, United States). Total RNA was reverse transcribed to cDNA using an RT reagent kit (Vazyme Biotech, Nanjing, China). The RT‐qPCR was performed using a SYBR Green assay (Vazyme Biotech, Nanjing, China) on a StepOnePlus real‐time PCR instrument (Thermo Fisher Scientific Inc., United States). The mRNA expression level of BMAL1, NCOA2, NOS2, NR1H3, SIAH2, and ADRB1 was normalized with ACTB, and the data were calculated through the 2^−ΔΔCt^ method. The primer sequences are listed in Table 1.

**Table 1:** Table of primer sequences.

**Gene (human)**	**Primer A**	**Primer B**
ACTB	AAAGCGGCTGTTAGTCACTGG	CGAGTCATTGCATACTGTCCAT
ADRB1	ATCGAGACCCTGTGTGTCATT	GTAGAAGGAGACTACGGACGAG
BMAL1	AAGGGAAGCTCACAGTCAGAT	GGACATTGCGTTGCATGTTGG
CLOCK	TGCGAGGAACAATAGACCCAA	ATGGCCTATGTGTGCGTTGTA
EGR3	CCAACGACATGGGCTCCATT	GGTCTCCAGAGGGGTAATAGG
GPR157	CATCCCGCTCATCTTCATCGG	CACCTCCCTGAAACGTGTTCC
NCOA2	CGAGGCGGAACAGCCATAC	GAGACAGCGAAGCACTGCATA
NOS2	CGAGGCGGAACAGCCATAC	CGAGGCGGAACAGCCATAC
NR1H3	CCTTCAGAACCCACAGAGATCC	ACGCTGCATAGCTCGTTCC
OPN4	CTGGATCTCCATACGGAGGC	GTAGAGGGACCGCCCATTT
RELB	CAGCCTCGTGGGGAAAGAC	GCCCAGGTTGTTAAAACTGTGC
SIAH2	TCTTCGAGTGTCCGGTCTG	CGGCATTGGTTACACACCAG
TH	TCTTCGAGTGTCCGGTCTG	CGGCATTGGTTACACACCAG
ZPBP2	GTAGTACGTCTGGATAGCTGTCG	CGCAGGTCTGACAAGTTACAT
ZFHX3	TGCAACCTCCAAACGCATCT	GGGACACTGGTACATCTGCTC

### 2.12. Statistical Analysis

Data were analyzed using R software. For forest plots, the hazard ratio (HR) was generated by univariate Cox or multivariate Cox proportional hazard regression, and the Kaplan–Meier approach was used for survival analyses. Differences between groups were assessed using the Wilcoxon test. Statistically significant differences were shown as follows:  ^∗^ *p* < 0.05,  ^∗∗^ *p* < 0.01,  ^∗∗∗^ *p* < 0.001, and NS, not significant.

## 3. Results

### 3.1. Identification of Circadian Rhythm Subtypes in Breast Cancer

Based on a cohort of 935 breast cancer samples with complete survival information (integrated from TCGA, METABRIC, and GEO series datasets), a consensus clustering algorithm was employed to perform pattern recognition on the expression profiles of 200 circadian rhythm genes. The CDF curves indicated optimal clustering stability at *k* = 3 (Figure 1a). Unsupervised clustering successfully categorized the samples into three subtypes with distinct characteristics (Figure 1b), and PCA further confirmed substantial transcriptomic differences among the subtypes (Figure 1c).

Figure 1Unsupervised clustering of circadian rhythm–related genes in BRCA. (a, b) BRCA samples were clustered by the consensus clustering method. (c) Visualization of consensus clustering results using PCA. (d–f) Kaplan–Meier curves showing overall survival in different circadian subtypes in the TCGA, METABRIC, and GSE42568 cohorts (log‐rank test). (g) Heatmap analysis showed the expression of circadian rhythm–related genes in three clusters. (h–j) Landscape of genomic aberrations of different clusters.

**Figure:**
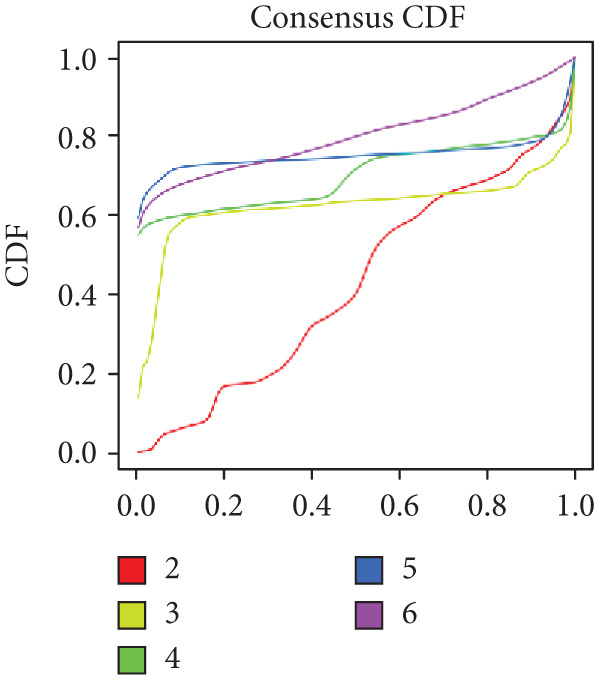
(a)

**Figure:**
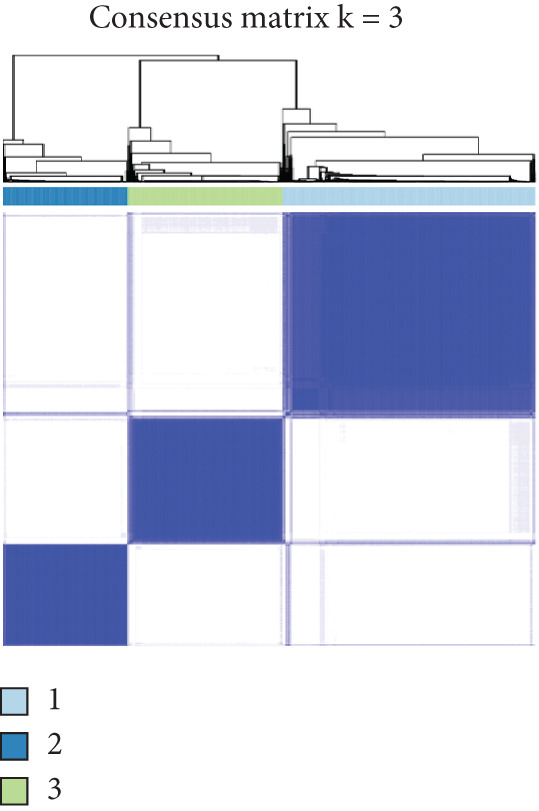
(b)

**Figure:**
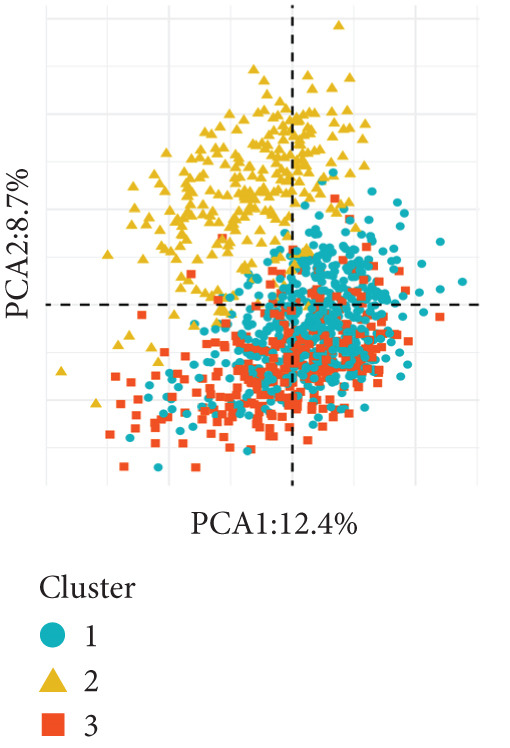
(c)

**Figure:**
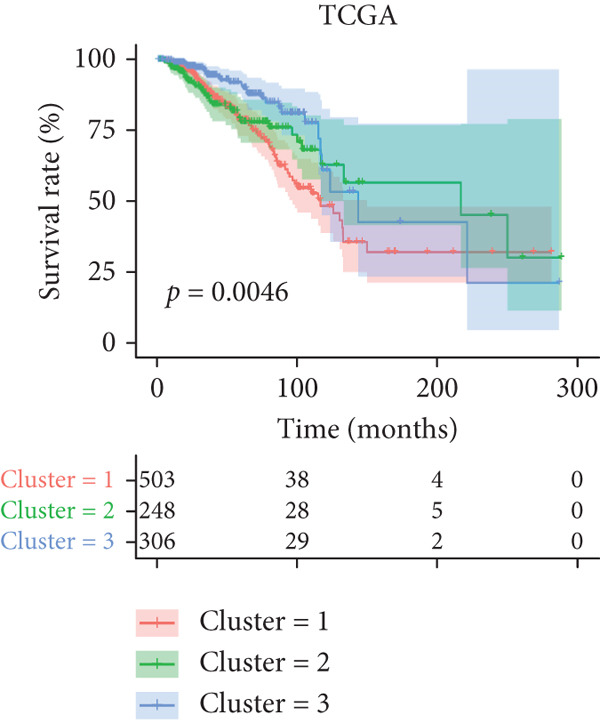
(d)

**Figure:**
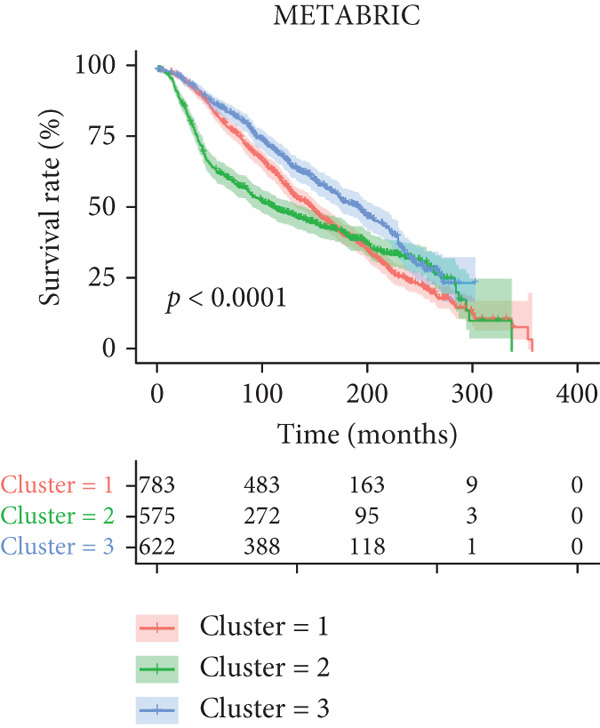
(e)

**Figure:**
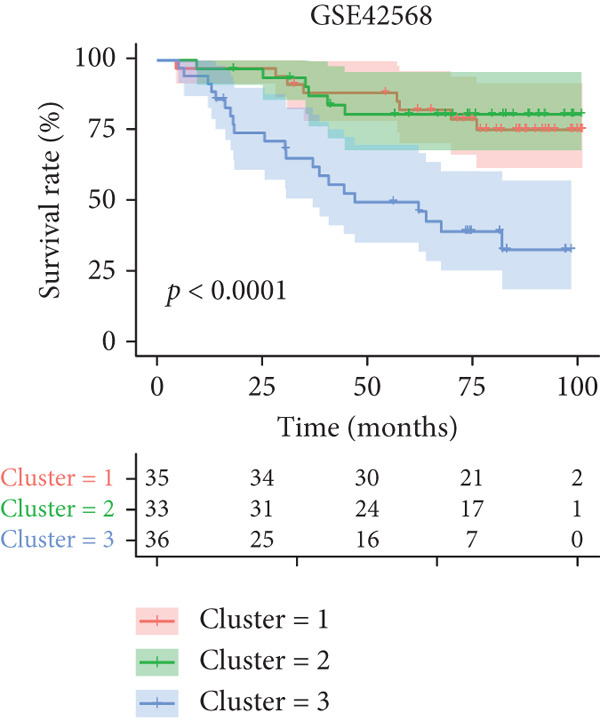
(f)

**Figure:**
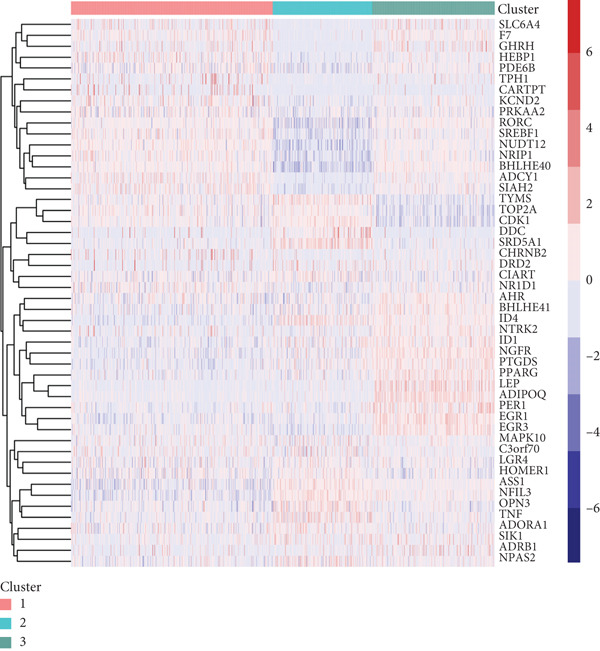
(g)

**Figure:**
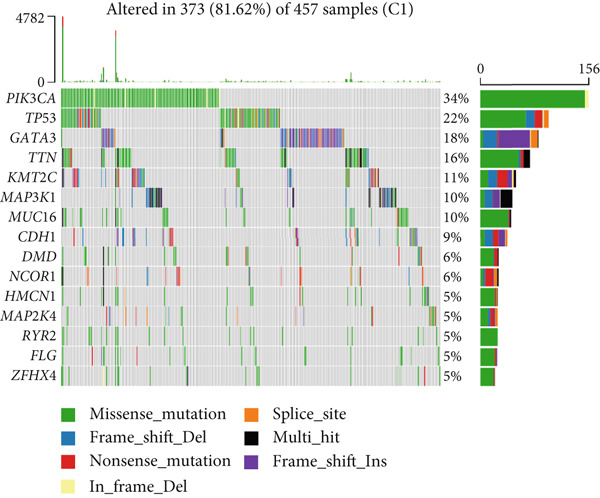
(h)

**Figure:**
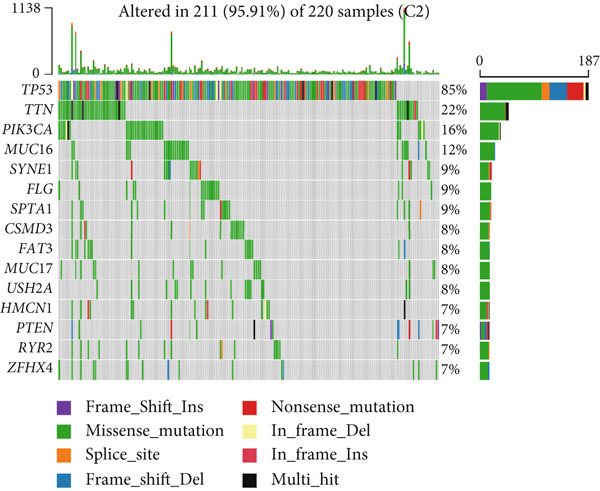
(i)

**Figure:**
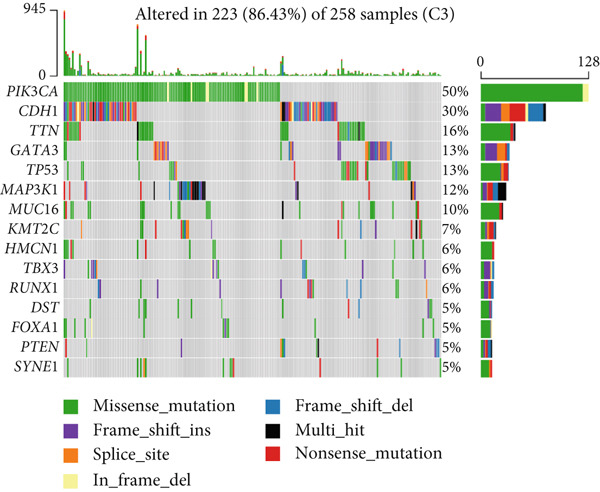
(j)

Multicenter survival analysis (conducted across TCGA, METABRIC, GSE42568, GSE61304, GSE2034, and GSE21653 datasets) revealed significant prognostic differences among the subtypes: Patients in Cluster 1 had the shortest OS, while Cluster 3 demonstrated the most favorable survival time (Figures 1d, 1e, and 1f, Figures S1A–C).

To elucidate the molecular mechanisms by which CRD influences breast cancer progression, we conducted a DEG analysis. Heatmaps revealed distinct gene expression patterns across the subtypes (Figure 1g). Genomic variation analysis identified significant enrichment of TP53 mutations in Cluster 1, while PIK3CA mutations were more frequently observed in Cluster 3 (Figures 1h, 1i, and 1j). These findings demonstrate significant differential gene expression patterns among the circadian rhythm–related subtypes, providing a theoretical foundation for personalized treatment strategies tailored to the distinct circadian rhythm gene expression profiles of patients with breast cancer.

### 3.2. Molecular Characterization of Different Circadian Rhythm Subtypes

To investigate the characteristic differences among the subtypes, we compared multiple clinical and pathological features including ER receptor status, HER2 status, PR receptor status, TNM staging, pathological grade, luminal classification, tumor location, and patient age (Figures 2a, 2b, 2c, 2d, 2e, 2f, 2g, 2h, 2i, and 2j). The results revealed significant differences in the distribution of pathological stages across the three clusters. Specifically, pathologic T4 was most prevalent in Cluster 1, while pathologic T3 was relatively more common in Cluster 3 (*p* < 0.05). In terms of molecular subtypes, the basal subtype was most frequently observed in Cluster 2, whereas Luminal A was more prevalent in Cluster 3 (*p* < 0.0001).

Figure 2Clinical characteristic analysis of different circadian rhythm pattern subtypes. (a) The distribution of ER (estrogen receptor) in different clusters was shown. (b) The distribution of HER2 in different subtypes was shown. (c) The distribution of PR (progesterone receptor) in different clusters was shown. (d) The distribution of M stage in different clusters was shown. (e) The distribution of grade in different clusters was shown. (f) The distribution of T stage in different clusters was shown. (g) The distribution of N stage in different clusters was shown. (h) The distribution of subtypes of BRCA in different clusters was shown. (i) The distribution of location in different clusters was shown. (j) The distribution of age in different clusters was shown.  ^∗^ *p* < 0.05;  ^∗∗^ *p* < 0.01;  ^∗∗∗^ *p* < 0.001;  ^∗∗∗∗^ *p* < 0.0001.

**Figure:**
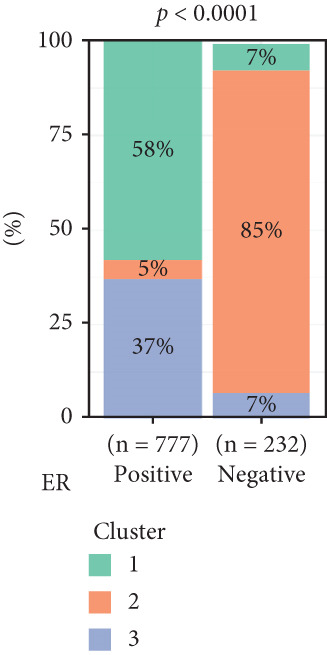
(a)

**Figure:**
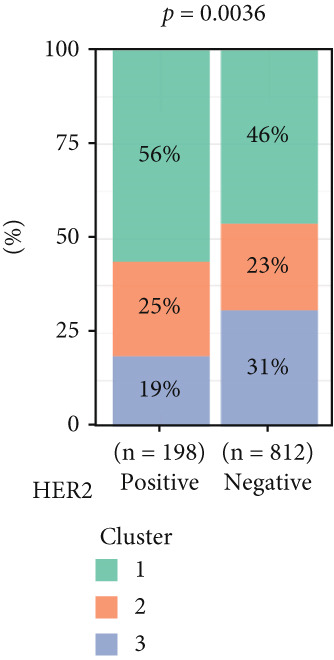
(b)

**Figure:**
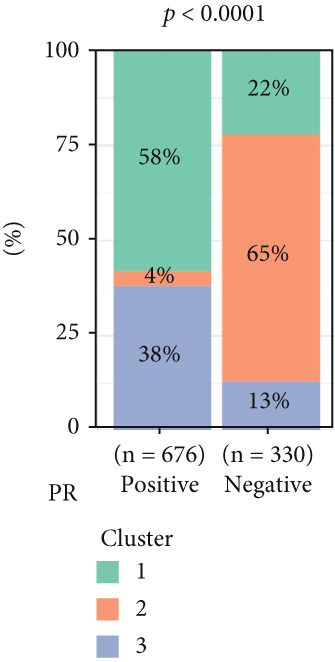
(c)

**Figure:**
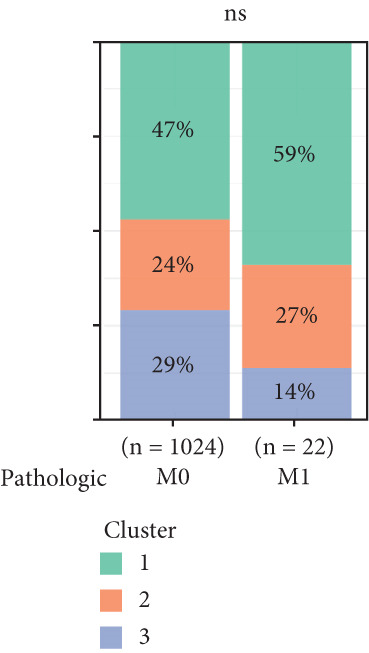
(d)

**Figure:**
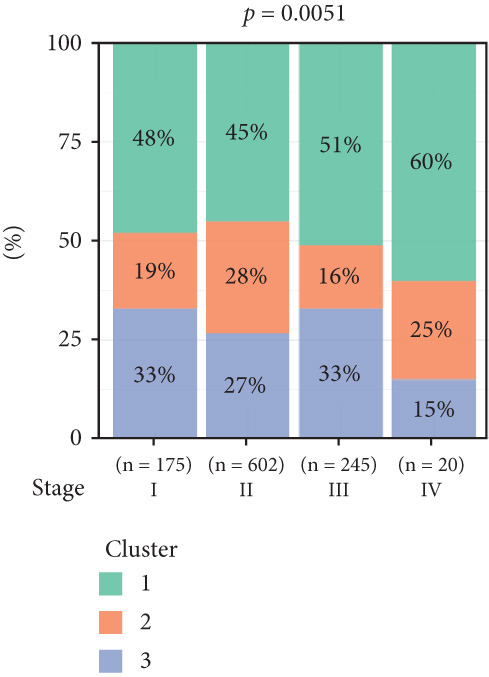
(e)

**Figure:**
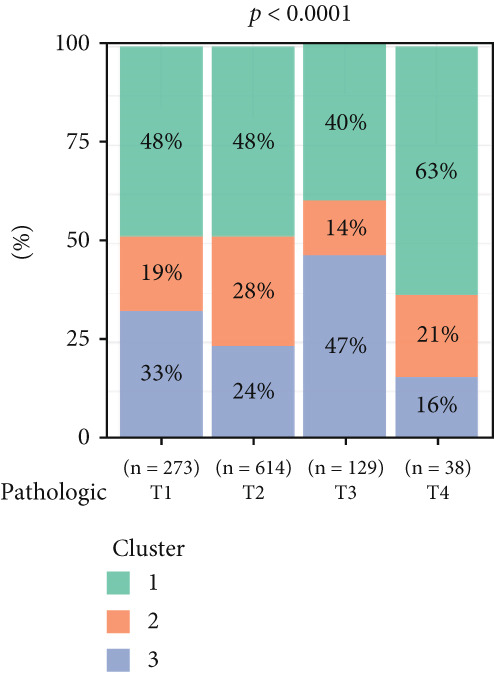
(f)

**Figure:**
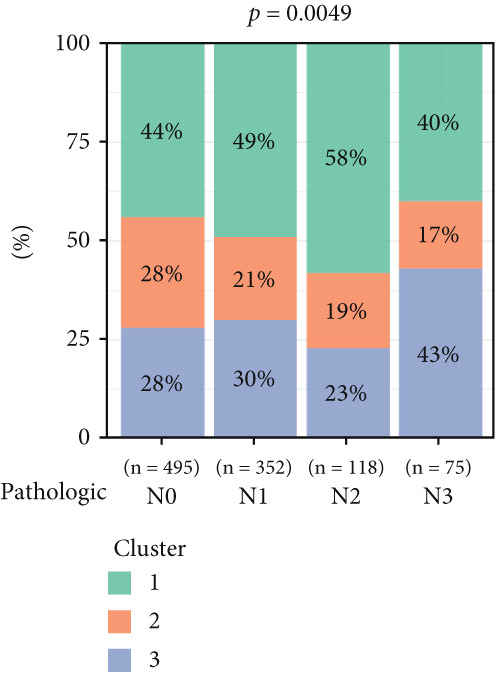
(g)

**Figure:**
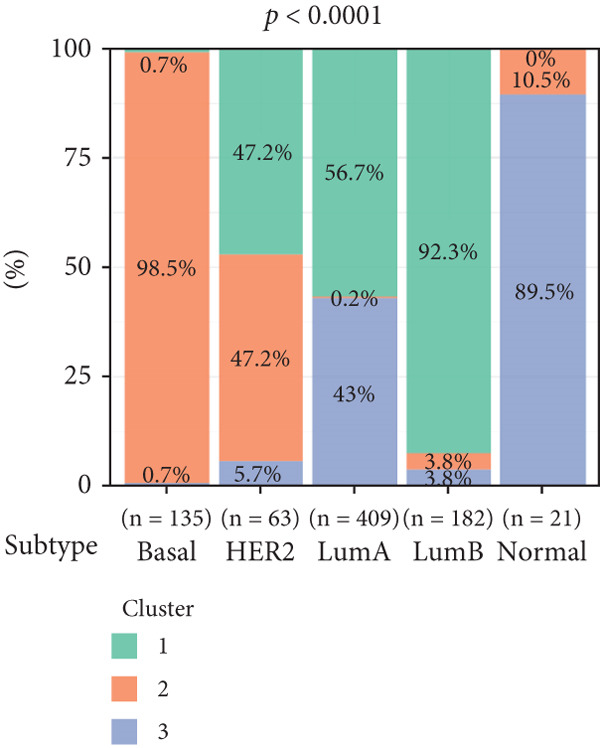
(h)

**Figure:**
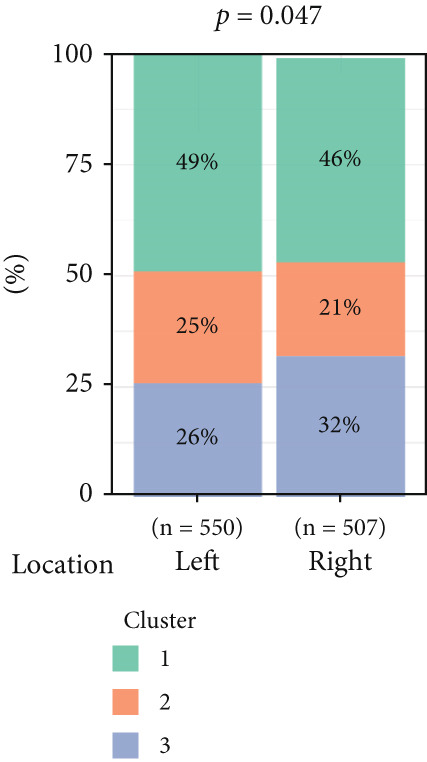
(i)

**Figure:**
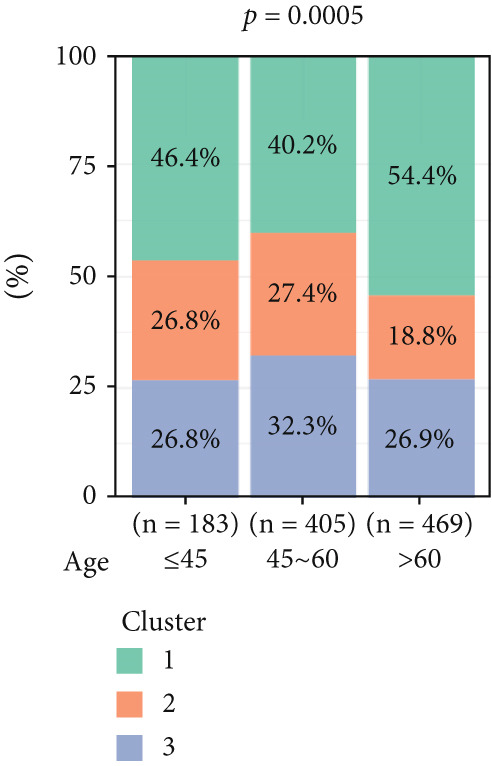
(j)

Age was closely associated with circadian rhythm subtypes. We found statistically significant differences in the distribution of age groups across the three clusters (*p* < 0.001). For instance, Cluster 1 had the highest proportion of patients aged > 60 years, while patients aged 45–60 were more common in Cluster 3. However, no significant differences were observed among the subtypes in terms of distant metastasis at diagnosis (Figure 2d).

### 3.3. Functional Enrichment Analysis of DEGs in Circadian Rhythm–Related Subtypes of Breast Cancer

To explore the potential mechanisms by which circadian rhythm–related genes influence breast cancer progression, we analyzed DEGs among the subtypes. The limma algorithm was used to identify DEGs between BRCA and normal samples with a filtering threshold of FDR *q*‐value more than 0.05 and log2 (|FC|) more than 1.5; the results revealed a total of 2016 DEGs in Cluster 1 compared to Cluster 2, 501 DEGs in Cluster 1 compared to Cluster 3, and 1904 DEGs in Cluster 2 compared to Cluster 3 (Figure 3a). By identifying the intersection of these DEGs, we screened out 52 core DEGs (Figure 3b). To further determine their biological functions, we performed KEGG pathway enrichment analysis using DAVID. As shown in Figure 3d, the upregulated genes in Cluster 1 were significantly enriched in pathways such as thyroid hormone synthesis, serotonergic synapse, pancreatic secretion, and oxytocin signaling (Figure 3c). The upregulated genes in Cluster 2 were significantly associated with pathways including viral protein interaction with cytokine and cytokine receptors, protein digestion and absorption, progesterone‐mediated oocyte maturation, and phototransduction. In Cluster 3, the upregulated genes were notably enriched in pathways such as viral protein interaction with cytokine and cytokine receptors, tyrosine metabolism, *Staphylococcus aureus* infection, and renin secretion (Figure 3e).

Figure 3Differential gene expression analysis and functional enrichment between BRCA clusters in the TCGA cohort. (a) Volcano plot of differentially expressed genes between Cluster A, Cluster B, and Cluster C. (b) Venn plot of the intersection of upregulated differentially expressed genes and selected genes from WGCNA. (c) The top 20 GO functions enriched for the upregulated 2016 genes. (d) The top 20 GO functions enriched for the upregulated 1904 genes. (e) The top 21 GO functions enriched for the upregulated 501 genes.

**Figure:**
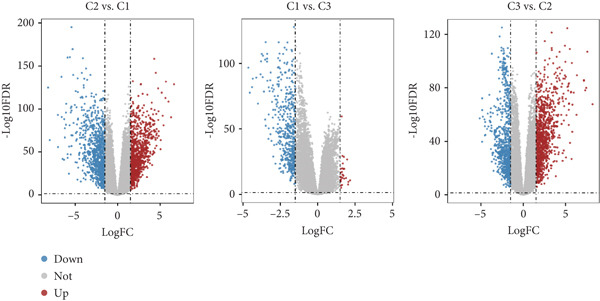
(a)

**Figure:**
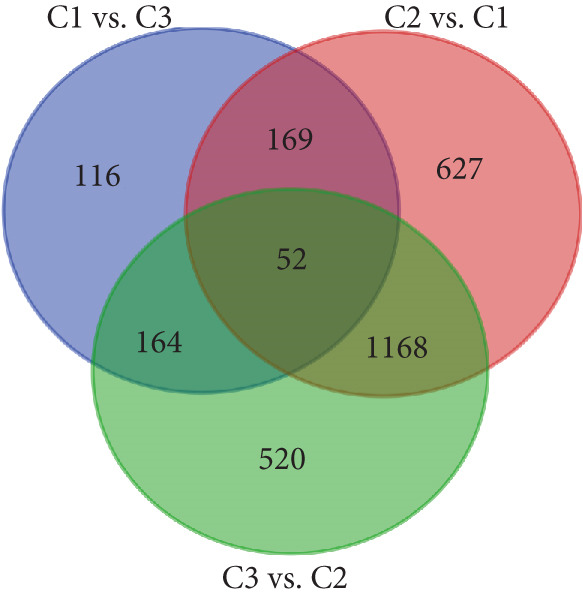
(b)

**Figure:**
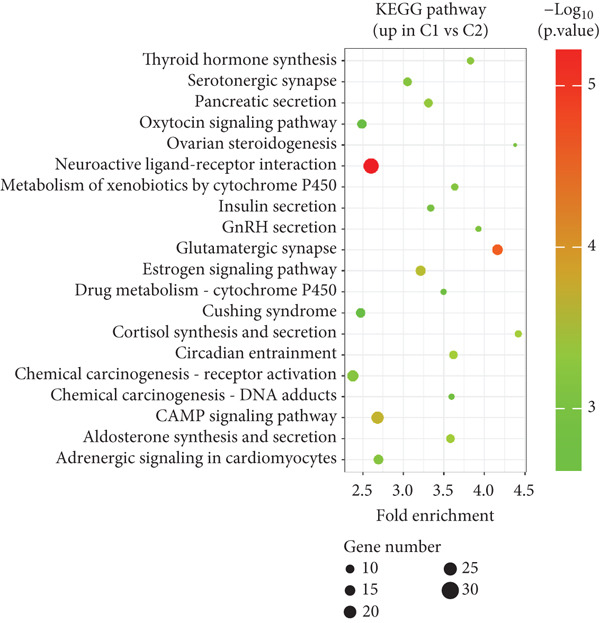
(c)

**Figure:**
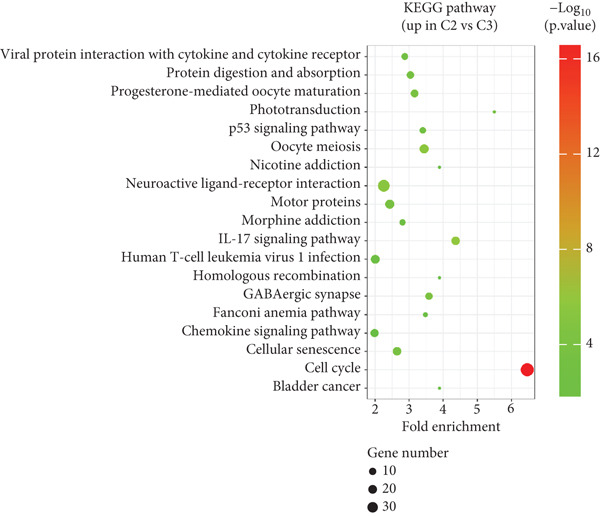
(d)

**Figure:**
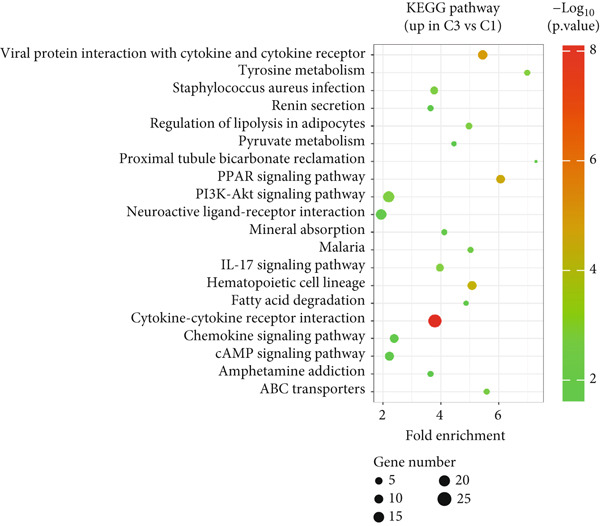
(e)

### 3.4. Circadian Rhythm–Related Gene‐Based Subtypes Exhibit Distinct Immune Characteristics

Given that immune cell infiltration is a critical factor influencing tumor progression and response to immunotherapy, we assessed the differences in tumor microenvironment (TME) immune cell infiltration between the subtypes. We found that patients in Cluster 1 had significantly lower immune scores compared to the other subtypes but exhibited the highest tumor cell purity, while Cluster 3 showed the highest stromal scores (Figure 4a). Specifically, Cluster 1 demonstrated lower infiltration scores for T cells, CD8 T cells, cytotoxic lymphocytes, B‐lineage cells, NK cells, monocytic lineage cells, myeloid dendritic cells, and endothelial cells, but significantly elevated fibroblast activity, suggesting enhanced stromal remodeling capacity. In contrast, Cluster 3 exhibited slightly higher infiltration scores for NK cells and B‐lineage cells than other subtypes, along with increased endothelial cell scores, potentially indicating angiogenesis‐related activity (Figure 4b).

Figure 4Immune characteristics of different circadian rhythm pattern subgroups. (a) Box plots of the immune, stromal, and tumor purity score between different subtypes. (b) Relative proportions of 10 immune cells in different subgroups. (c) Gene expression of immune checkpoints between three distinct clusters. (d, e) Violin plot of the TMB and TIDE score between three subtypes.  ^∗^ *p* < 0.05;  ^∗∗^ *p* < 0.01;  ^∗∗∗^ *p* < 0.001;  ^∗∗∗∗^ *p* < 0.0001.

**Figure:**
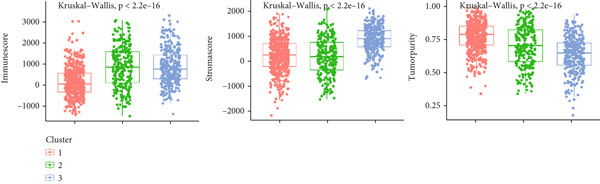
(a)

**Figure:**
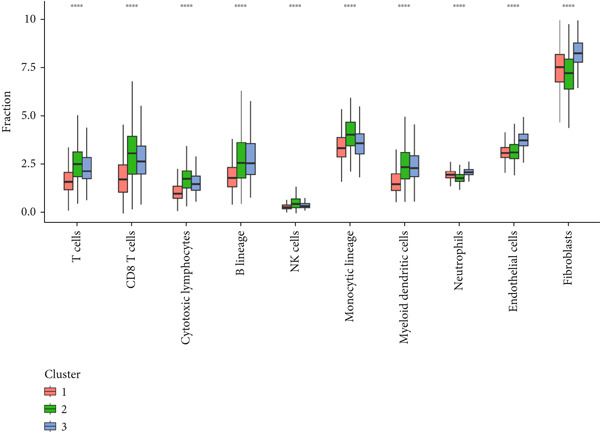
(b)

**Figure:**
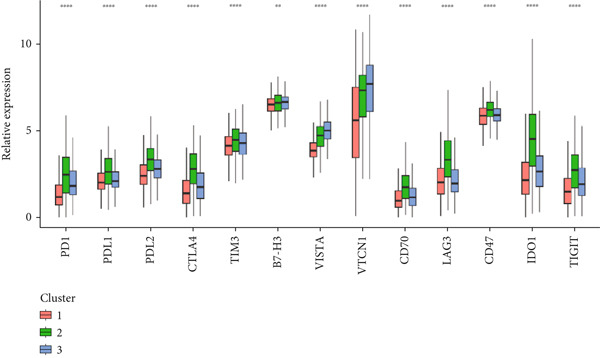
(c)

**Figure:**
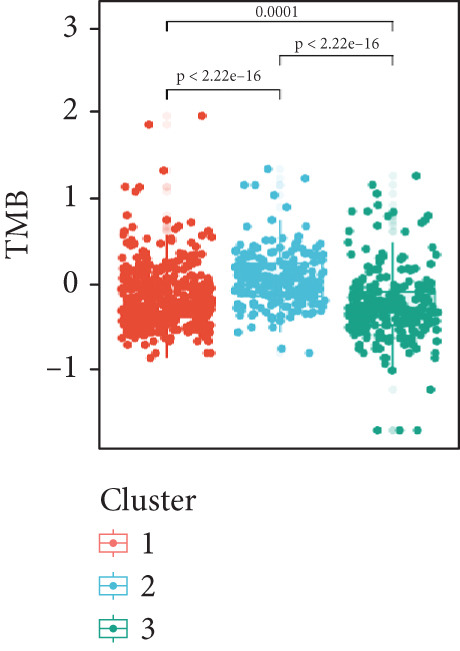
(d)

**Figure:**
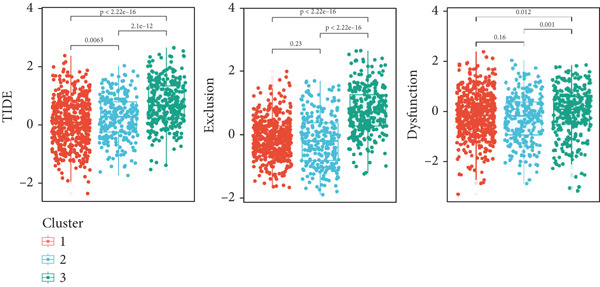
(e)

Notably, immune checkpoints (e.g., PD1, PDL1, and CTLA4) were significantly upregulated in Cluster 2 and Cluster 3, while their expression was lower in Cluster 1 (Figure 4c). Additionally, Cluster 1 had higher tumor mutational burden (TMB) scores and lower TIDE, exclusion, and dysfunction scores, suggesting greater potential for immunotherapy responsiveness. Conversely, Cluster 2 and Cluster 3 exhibited lower TMB scores and higher TIDE scores, indicating possible tumor immune escape mechanisms (Figure 4d).

### 3.5. Molecular Subtypes Based on Circadian Rhythm–Related Genes Predict Drug Response in Breast Cancer

We analyzed the differences in drug sensitivity among the subtypes and evaluated the relationship between drug sensitivity (measured by IC_50_ values) using Spearman correlation analysis (Figure 5a). The results revealed that Cluster 1 exhibited sensitivity to nearly all hormonal, targeted, and chemotherapeutic agents, with the most notable responses observed for hormonal therapy and CDK4/6 inhibitors. Cluster 2 showed the poorest response to chemotherapy and endocrine therapy, suggesting that CDK4/6 inhibitors (e.g., ribociclib and palbociclib) and PARP inhibitors (e.g., olaparib and talazoparib) may be appropriate treatment options in this group. Cluster 3 demonstrated intermediate sensitivity, with AKT inhibitors (ipatasertib), HDAC inhibitors (entinostat), and hormonal therapies (tamoxifen and fulvestrant) emerging as potentially effective treatment options. These findings suggest that tailored drug selection should be considered for breast cancer patients based on their specific circadian rhythm–related subtypes.

Figure 5Drug response analysis in different clusters. Wilcoxon test. (a) The differences in the chemotherapy response of common chemotherapy drugs in three groups. (b) The correlation analysis between drug sensitivity (IC_50_ values) and gene expression levels.

**Figure:**
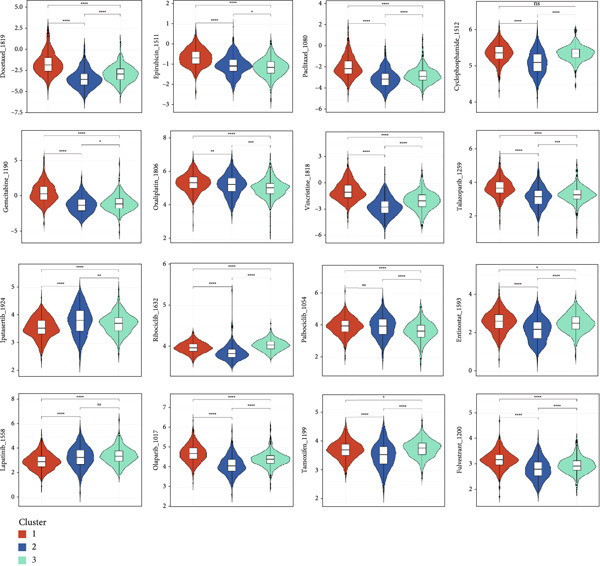
(a)

**Figure:**
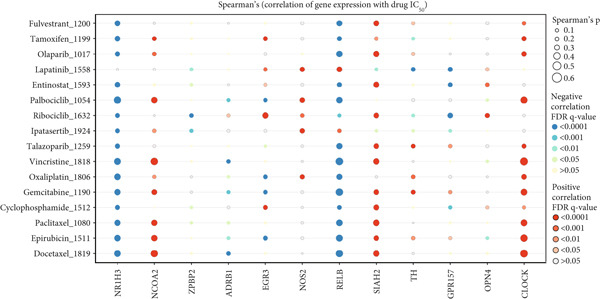
(b)

### 3.6. Circadian Rhythm–Related Gene Signatures as Predictive Tools for Breast Cancer Prognosis

**Figure 6:**
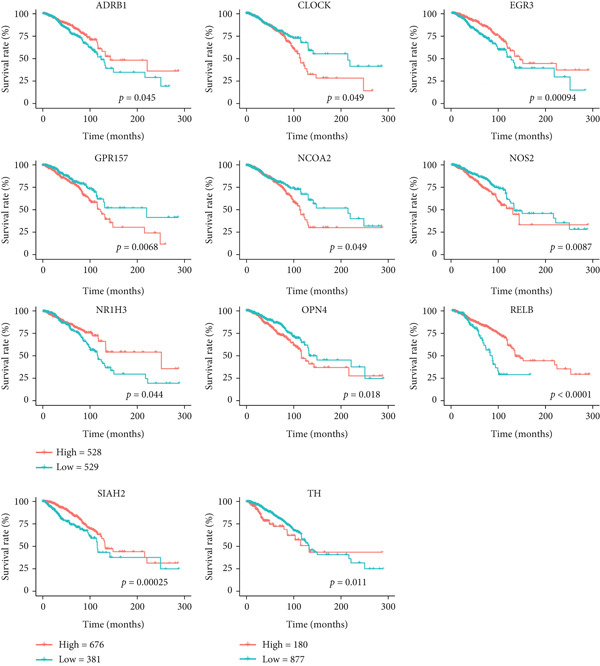
The correlation between circadian rhythm–related gene expression and overall survival time in BRCA was analyzed, including ADRB1, CLOCK, EGR3, GPR157, NCOA2, NOS2, NR1H3, OPN4, RELB, SIAH2, and TH.

To evaluate the predictive value of circadian rhythm–related genes for breast cancer prognosis, we analyzed the correlation between gene expression and patient OS. The results indicated that high expression levels of OPN4, NOS2, GPR157, NCOA2, CLOCK, and TH were associated with shorter OS in breast cancer patients, while high expression of EGR3, NR1H3, RELB, SIAH2, and ADRB1 was linked to improved survival rates (Figure 6).

Additionally, we employed Spearman correlation analysis to investigate the relationship between gene expression levels and IC_50_ values. Heatmaps and scatter plots revealed that high expression of genes such as REL, SIAH2, and EGR3 may enhance the efficacy of chemotherapeutic agents (e.g., docetaxel, epirubicin, paclitaxel, and oxaliplatin) and PARP inhibitors (e.g., talazoparib). In contrast, high expression of OPN4, ZBPB2, NOS2, and CLOCK was associated with resistance to docetaxel, epirubicin, and paclitaxel, while NR1H3 and NCOA2 expression may influence the effectiveness of endocrine therapies (e.g., tamoxifen and fulvestrant) (Figure 5b).

### 3.7. Construction of a Circadian Rhythm–Related Risk Model

We integrated survival outcomes from the TCGA, METABRIC, GSE42568, GSE61304, GSE2034, and GSE21653 cohorts. As shown in Figure 7a, 13 genes were observed to influence breast cancer survival outcomes, among which ADRB1, EGR3, NR1H3, RELB, and SIAH2 were protective factors, while the remaining genes were significant risk factors. Using LASSO‐penalized Cox regression analysis, we screened 12 key genes from the DEGs to construct a CRD risk score (Figure 7b,c). Ultimately, 12 circadian rhythm–related genes were selected and incorporated to establish a novel risk‐scoring system.

Figure 7Developed nomogram to predict the probability of survival in BRCA patients. (a) Univariate Cox regression analyses of risk scores in multiple independent cohorts. (b, c) Circadian rhythm–related risk score models were constructed using least absolute shrinkage and LASSO methods. (d) LASSO coefficients of the 14 circadian rhythm–related genes. (e, f) The association of high‐risk score with prognosis of BRCA patients. (g) ROC curves for predicting 1‐, 5‐, and 10‐year OS by risk score. (h) Prognostic nomogram Cox regression analyses of risk scores in multiple independent cohorts. (i) Time‐dependent ROC curves at 1, 3, and 5 years. (j) Line graph of the area under the curve.

**Figure:**
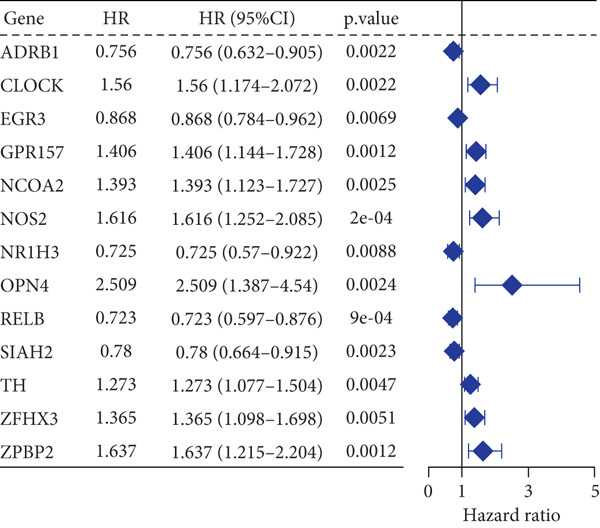
(a)

**Figure:**
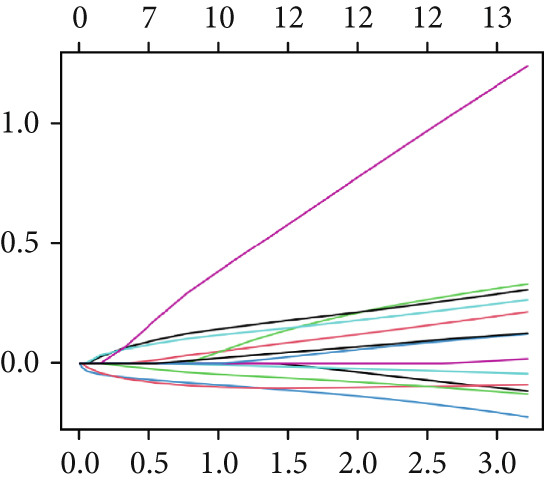
(b)

**Figure:**
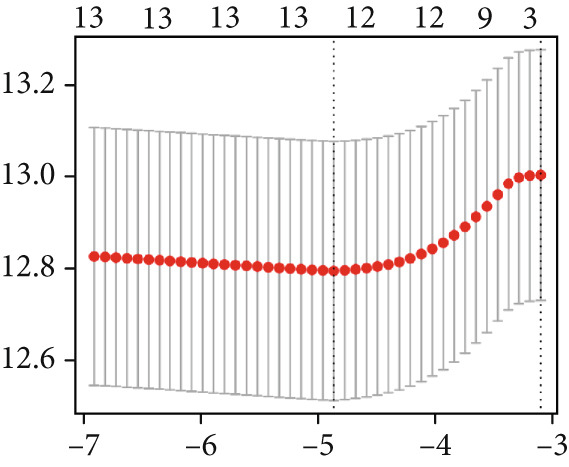
(c)

**Figure:**
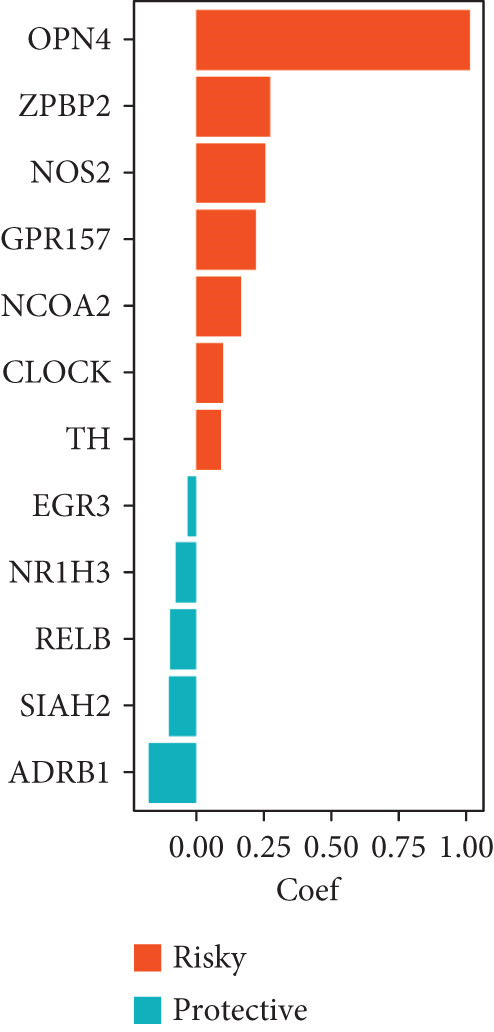
(d)

**Figure:**
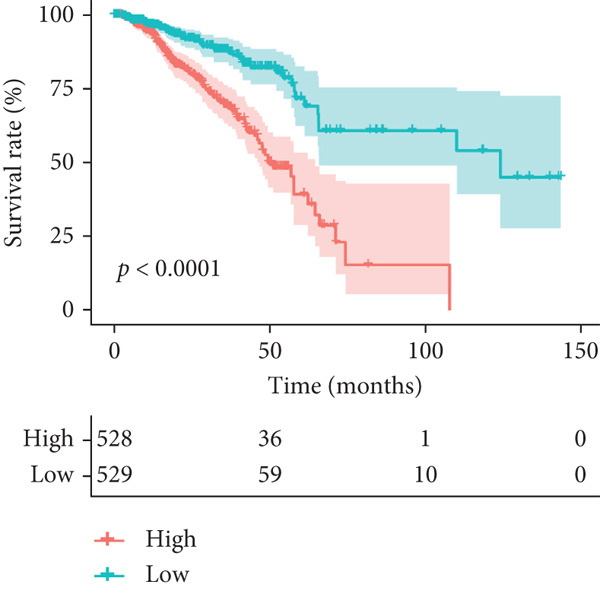
(e)

**Figure:**
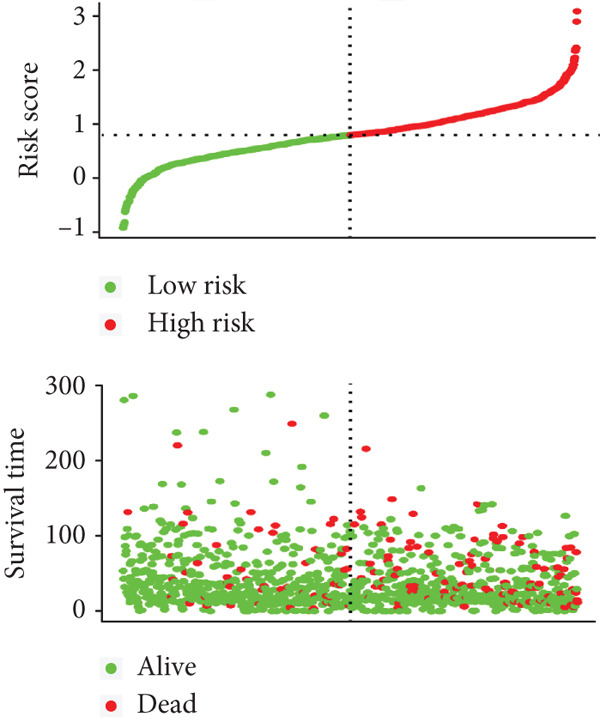
(f)

**Figure:**
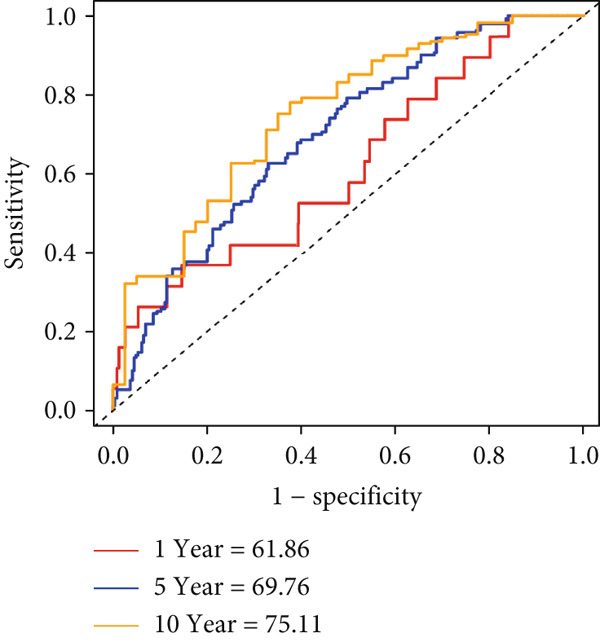
(g)

**Figure:**
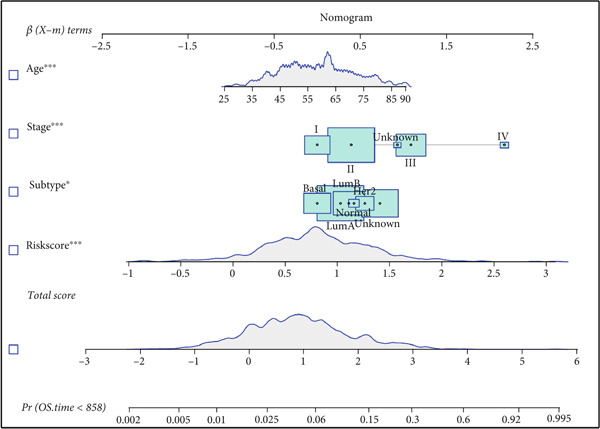
(h)

**Figure:**
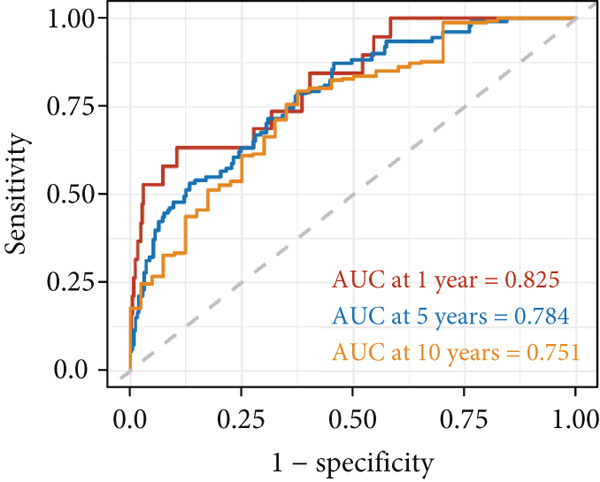
(i)

**Figure:**
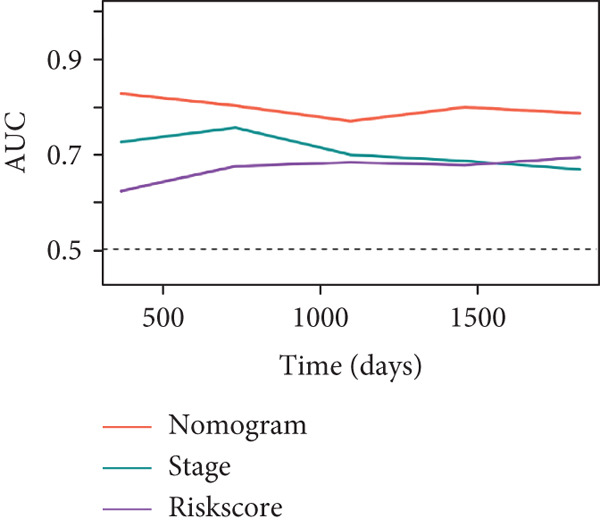
(j)

Based on the expression levels of these 12 genes, we calculated the CRD risk signature score and stratified patients into high‐risk and low‐risk groups (Figure 7f). Kaplan–Meier survival analysis and univariate Cox regression analysis revealed that patients in the low‐risk group had a significant survival advantage compared with those in the high‐risk group (Figure 7e, Figure S4A). The Wilcoxon test further confirmed that the risk score effectively distinguished between surviving and deceased patients, with the high‐risk group exhibiting significantly higher mortality (Figure S4B). According to the ROC curves, the CRD risk score in our current study demonstrated strong performance in medium‐ and long‐term (5 years and 10 years) survival prediction (Figure 7g, Figure S4C).

Additionally, we constructed a nomogram by combining the risk score with clinicopathological characteristics (age, stage, and subtype) (Figure 7h). This model showed excellent predictive performance, with AUC values of 0.825, 0.784, and 0.751 for 1‐year, 5‐year, and 10‐year RFS prediction, respectively (Figure 7i). Comparative analysis revealed that the integrated model outperformed individual staging and risk score models at all time points, particularly in short‐term prediction (500 days, AUC > 0.85) (Figure 7j).

### 3.8. Validation of Circadian Rhythm–Related Genes

**Figure 8:**
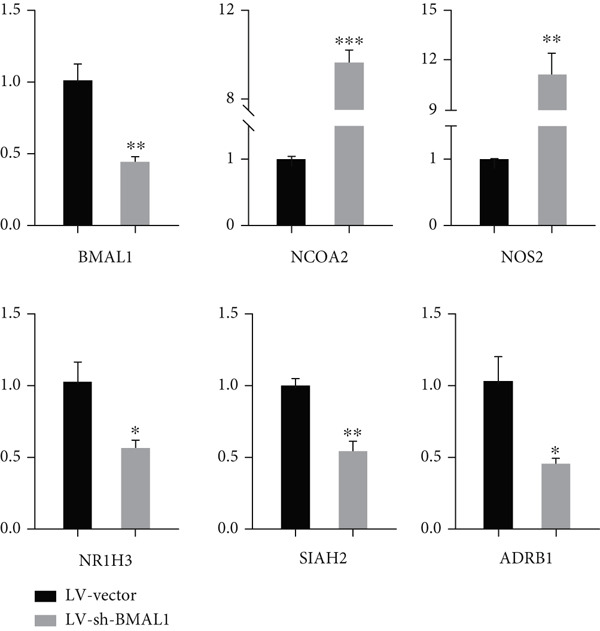
Validation of circadian rhythm–related genes, including BMAL1, NCOA2, NOS2, NR1H3, SIAH2, and ADRB1.

Through lentivirus‐mediated shRNA technology, we established stable BMAL1 knockdown in human breast cancer cell lines (SKBR3). RT‐qPCR analysis confirmed a significant reduction of BMAL1 mRNA levels in shBMAL1 groups compared with shVector controls (Figure 8). Subsequent detection of circadian rhythm–related genes revealed elevated risk‐associated factors and decreased protective factors (Figure 8), consistent with findings in Figure 7d. These data suggest that CRD regulates the transcriptional activity of circadian rhythm–related genes in breast cancer.

## 4. Discussion

In summary, based on the expression levels of 200 circadian rhythm–related genes, we identified three circadian rhythm–associated breast cancer subtypes, which exhibited significant differences in prognosis, clinical characteristics, and molecular features. Among these, Cluster 1 demonstrated the lowest survival rates, suggesting that this subtype may represent a subgroup with a poorer prognosis. Conversely, Cluster 3 consistently showed the highest survival rates, indicating that these patients may have more favorable clinical outcomes in the real world. However, the results differed in the GSE42568 dataset, where Cluster 3 exhibited the lowest survival rates, while Cluster 2 had the best prognosis. This discrepancy may be attributed to the smaller sample size of the GSE42568 dataset (approximately 30 cases per group) or tumor heterogeneity. Therefore, future studies using larger datasets are needed to validate these findings.

Gene mutation signature analysis revealed that missense mutations were the most predominant type across all categories. PIK3CA emerged as the primary driver gene in Clusters 1 and 3, with mutations leading to an abnormal activation of the PI3K pathway, thereby promoting cell proliferation [13]. In contrast, TP53 exhibited a mutation rate of up to 85% in Cluster 2, playing a central role in cell cycle regulation and DNA damage repair [14]. These differences in mutation profiles may serve as a foundation for molecular subtyping, aiding in the guidance of precision therapy.

Clinical feature analysis demonstrated that Cluster 1 was significantly associated with Luminal A and Luminal B subtypes, primarily observed in hormone receptor–positive (ER+ and PR+) patients, which typically indicate a more favorable prognosis [15]. This cluster showed a high proportion in both early stage (Stage I) and late‐stage (Stage IV) patients. Cluster 2 was strongly correlated with the basal subtype and predominantly found in ER‐/PR‐ and HER2‐positive patients, suggesting higher aggressiveness and poorer prognosis [16, 17]. Cluster 3 showed a higher association with the Luminal A subtype and was more prevalent among patients with lymph node metastasis, indicating a potential link to metastatic behavior.

The inconsistency between these distribution patterns and the prognostic analysis results may stem from dataset heterogeneity or differences in treatment responses. For instance, the high sensitivity of Cluster 1 to endocrine therapy may be the key reason for improved prognosis, while the aggressive features of Cluster 2 might have been partially mitigated by targeted therapies. Therefore, future studies should focus on integrating the diversity of different datasets and individualized treatment regimens to achieve more accurate prediction of patient prognosis and provide more effective guidance for precision therapy.

Differential gene analysis revealed that Cluster 2 exhibited the most significant differences compared to other subtypes, reflecting its unique molecular characteristics. KEGG pathway enrichment analysis demonstrated that Cluster 1 was significantly enriched in estrogen signaling and steroid metabolism–related pathways, consistent with its hormone‐dependent nature. Cluster 2 showed significant enrichment in cell cycle and inflammation‐related pathways, supporting its highly proliferative and invasive biological behavior. Cluster 3 was notably enriched in metabolic pathways (e.g., fatty acid degradation and lipolysis regulation) and the PI3K/AKT signaling pathway, indicating metabolism as its key feature. These findings provide direction for subsequent experimental validation and target exploration.

Analysis of the tumor immune microenvironment revealed that Cluster 1 had the lowest TMB, indicating a lower genomic mutation load and weaker tumor immunogenicity [18]. Additionally, the expression of immune checkpoint molecules was lowest in Cluster 1, with reduced levels of inhibitory molecules such as PD1, PDL1, CTLA4, and LAG3, suggesting weaker immune evasion mechanisms [19]. Overall, Cluster 1 had the lowest TIDE score, fewer immune cell infiltrates, but intact immune cell functionality, and low immune checkpoint molecule expression, indicating potential high sensitivity to immunotherapy (e.g., PD1/PDL1 inhibitors) [20].

Cluster 2 exhibited the highest TMB and abundant immune cell infiltration, but severe immune cell exhaustion and significantly elevated immune checkpoint molecule expression, suggesting comprehensive activation of immune evasion mechanisms. Cluster 3 showed rich stromal components, the highest immune exclusion score, and the highest TIDE score, indicating potential strong resistance to immunotherapy [21].

Drug sensitivity analysis indicated that Cluster 1 was most sensitive to traditional chemotherapeutic agents (e.g., docetaxel, paclitaxel, epirubicin, and gemcitabine) and hormonal therapies (e.g., tamoxifen and fulvestrant), consistent with luminal subtype characteristics. Cluster 2 showed the highest sensitivity to targeted therapies (e.g., ipatasertib and ribociclib), suggesting that the PI3K/AKT and CDK4/6 pathways may be potential therapeutic targets. Cluster 3 exhibited the lowest sensitivity to traditional chemotherapy, targeted drugs, and hormonal therapies. High expressions of NR1H3, NCOA2, and REL were significantly associated with resistance to multiple chemotherapeutic agents, while CLOCK and SIAH2 expression positively correlated with drug sensitivity, indicating these genes as potential targets for overcoming drug resistance.

To quantify the expression levels of circadian rhythm–related genes in individual tumors, we developed a risk model based on circadian rhythm–related gene scoring, successfully stratifying patients into high‐risk and low‐risk groups. Subsequently, the model predicted significantly shorter survival times for high‐risk patients. To further enhance prediction accuracy, we constructed a nomogram by integrating the risk score with clinicopathological characteristics (age, stage, and subtype). This nomogram demonstrated excellent performance in predicting 1‐year, 5‐year, and 10‐year RFS (AUC values of 0.825, 0.784, and 0.751, respectively). In addition, the nomogram outperformed individual staging and risk score models at all time points, particularly for short‐term prediction (500 days, AUC > 0.85).

Although similar studies have been conducted using circadian genes, immune‐related genes, and glycosyltransferases to construct molecular subtypes and risk models [22–25], our study integrates circadian, immune, and metabolic signatures into a temporally resolved framework that predicts both therapeutic sensitivity and clinical outcomes. Compared to existing models, our nomogram offers superior practicality for resource‐limited settings and enables chronotherapy optimization. Our study bridges circadian biology, tumor immunology, and clinical oncology to deliver a precision medicine tool that is mechanistically insightful and immediately deployable.

Some limitations should be acknowledged in the present study. First of all, the predictive reliability and clinical application of the circadian risk signature need further validation in prospective clinical trials. Second, the predictive roles of circadian rhythm–related genes determined in our study need to be verified in larger clinical samples.

## 5. Conclusion

This study highlights the significant role of circadian rhythm–related molecular subtypes in breast cancer prognosis, immunotherapy response, and drug sensitivity and successfully establishes a prognostic model based on circadian rhythm genes. In addition, these findings provide potential therapeutic targets for the precise classification and personalized treatment of breast cancer. Future research should further integrate multiomics data and clinical treatment information to optimize model performance for real‐world application in clinical practice.

**Nomenclature**
CRD: circadian rhythm disruption
CRGs: circadian rhythm–related genes
CGS: circadian‐related gene signature
GEO: Gene Expression Omnibus
BRCA: breast cancer
ssGSEA: single‐sample gene set enrichment analysis
RFS: recurrence‐free survival
OS: overall survival
CDF: cumulative distribution function
PCA: principal component analysis
DEG: differential expression gene
TME: tumor microenvironment
TMB: tumor mutational burden
IC_50_
: half‐maximal inhibitory concentration

## Ethics Statement

The authors have nothing to report.

## Consent

The authors have nothing to report.

## Disclosure

All authors have read and agreed to the published version of the manuscript.

## Conflicts of Interest

The authors declare no conflicts of interest.

## Author Contributions

Lulu Tan and Yanzhen Lu: writing—original draft, conceptualization, methodology, data curation; Yunfei Yang and Dan Yu: methodology, formal analysis, data curation; Yuxi Tan: visualization, software, methodology; Gang Feng: software; Liquan Ouyang: formal analysis; Yuyan Tan: writing—review and editing, conceptualization. Lulu Tan and Yanzhen Lu contributed equally to this work.

## Funding

This study is funded by the Natural Science Foundation of Hubei Province (10.13039/501100003819, 2024AFB230).

## Supporting information


**Supporting Information** Additional supporting information can be found online in the Supporting Information section. Supporting figures: These figures include multicenter survival analysis, Kaplan–Meier survival analysis, and univariate Cox regression analysis.

## Data Availability

The gene expression profiles of BRCA tissues from four publicly available datasets (GSE42568, GSE61304, GSE2034, and GSE21653) were retrieved from the NCBI Gene Expression Omnibus (GEO) repository (https://www.ncbi.nlm.nih.gov/geo/). TCGA‐BRCA normalized data and clinical information were downloaded from the UCSC Xena website (https://xenabrowser.net). All gene expression profiles were normalized by R software.
