# Supplementary material for: Deciphering Circadian Rhythm–Related Molecular Subtypes in Breast Cancer and Establishing a Prognostic Prediction Model
Source: Biomed Res Int. 2025 Sep 9;2025:9664238. doi: 10.1155/bmri/9664238 (PMC12419142; doi:10.1155/bmri/9664238)
Supplement: Supplementary file 1 — Supporting Information Additional supporting information can be found online in the Supporting Information section. Supporting figures: These figures include multicenter survival analysis, Kaplan–Meier survival analysis, and univariate Cox regression analysis. [file BMRI-2025-9664238-s001.docx]

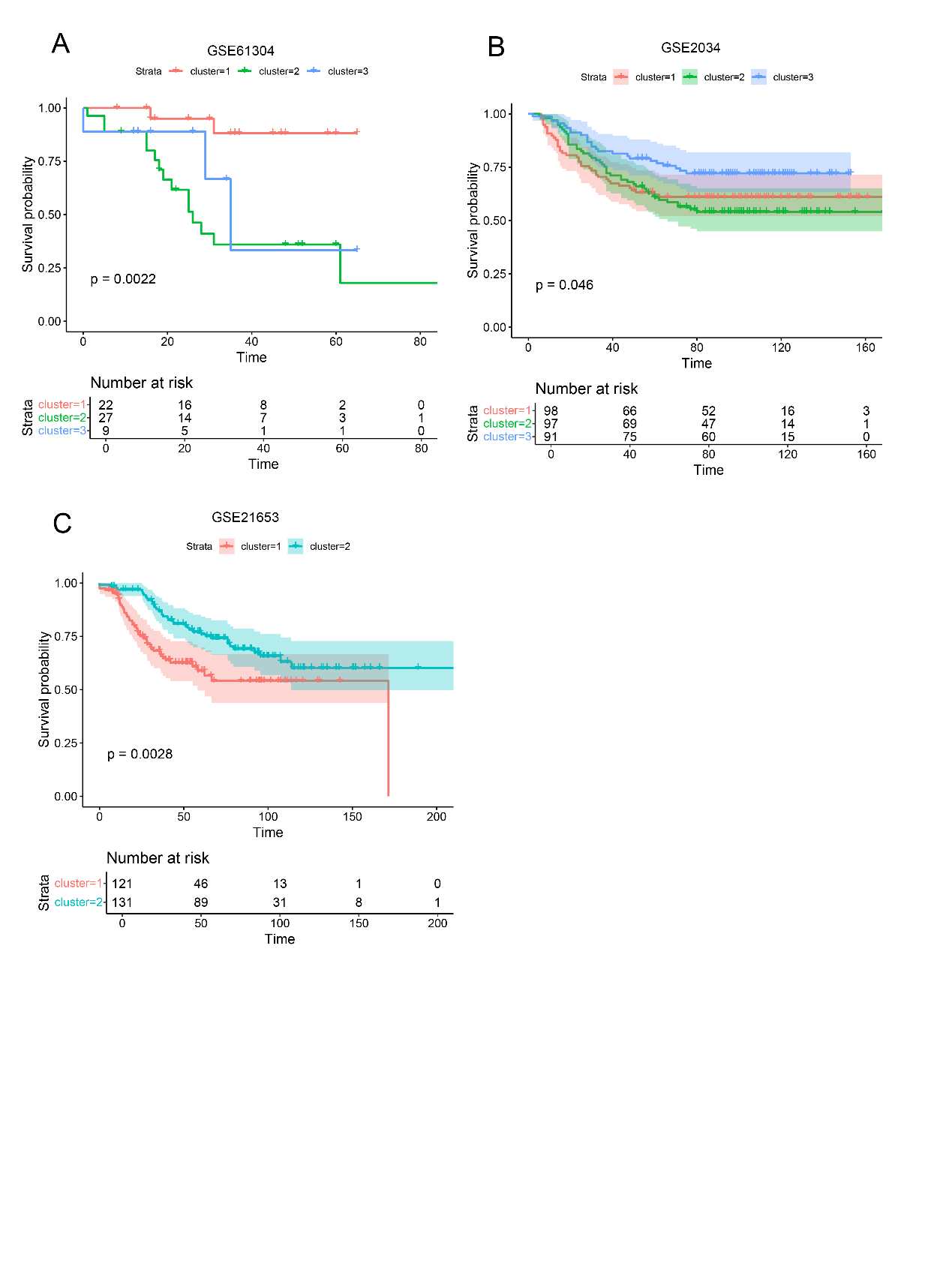


**Supplementary Figures 1.** Kaplan–Meier curves showing overall survival in different circadian subtypes in the GSE61304, GSE2034 and GSE21653 cohorts (log-rank test).


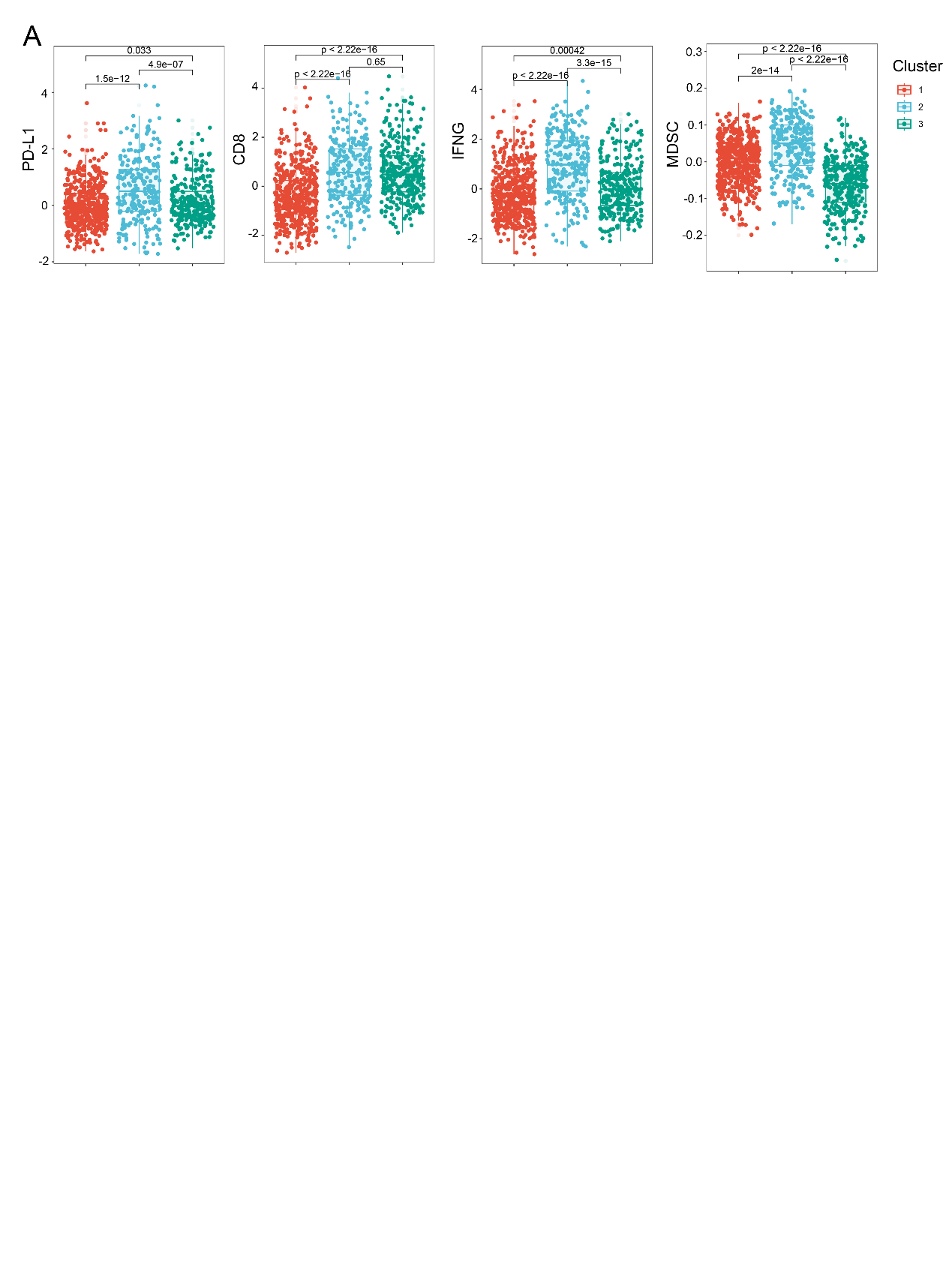


**Supplementary Figures 2.** Gene expression of immune checkpoints between three distinct clusters.


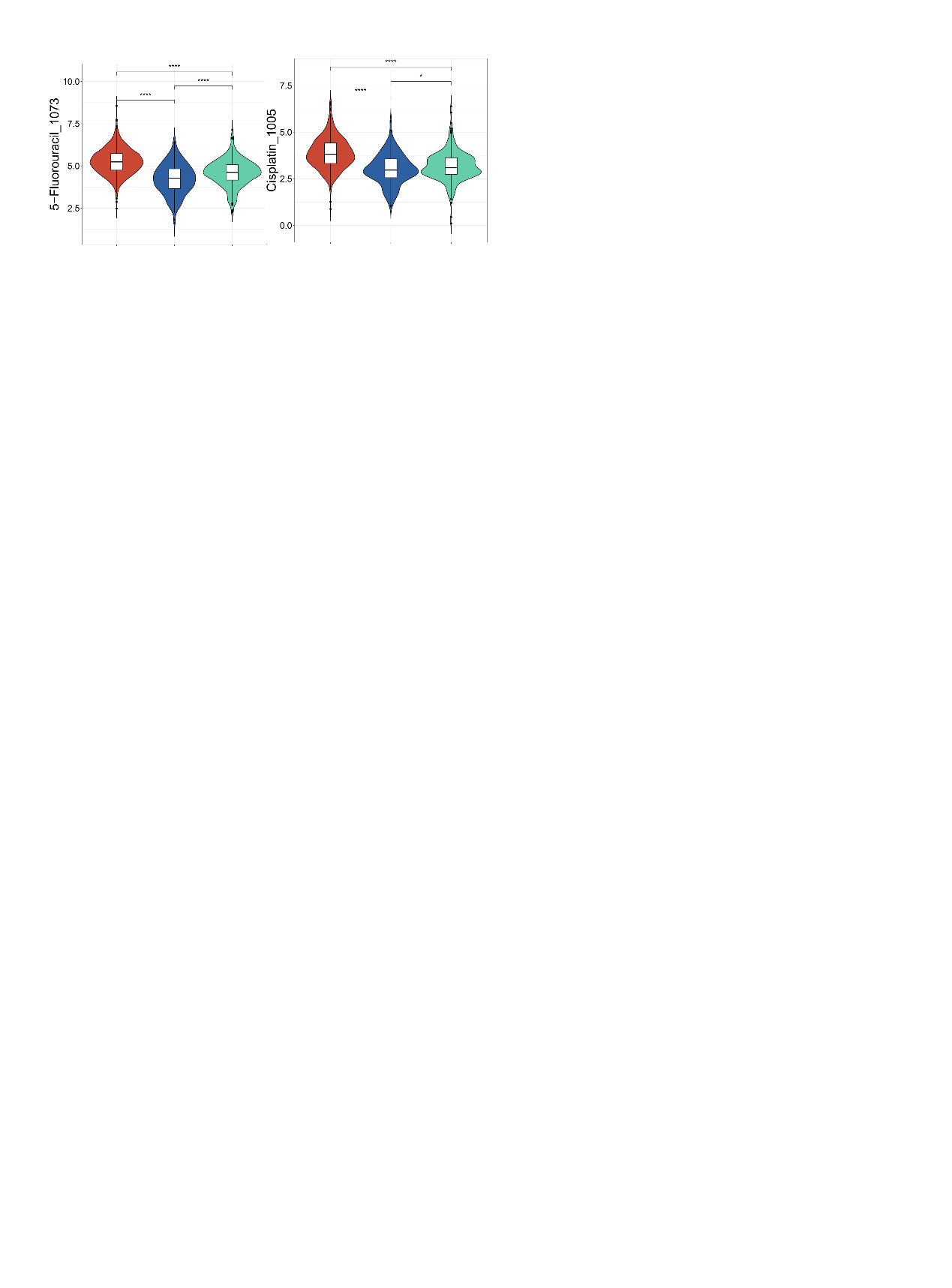


**Supplementary Figures 3.** The differences in the chemotherapy response of common chemotherapy drugs in three groups.


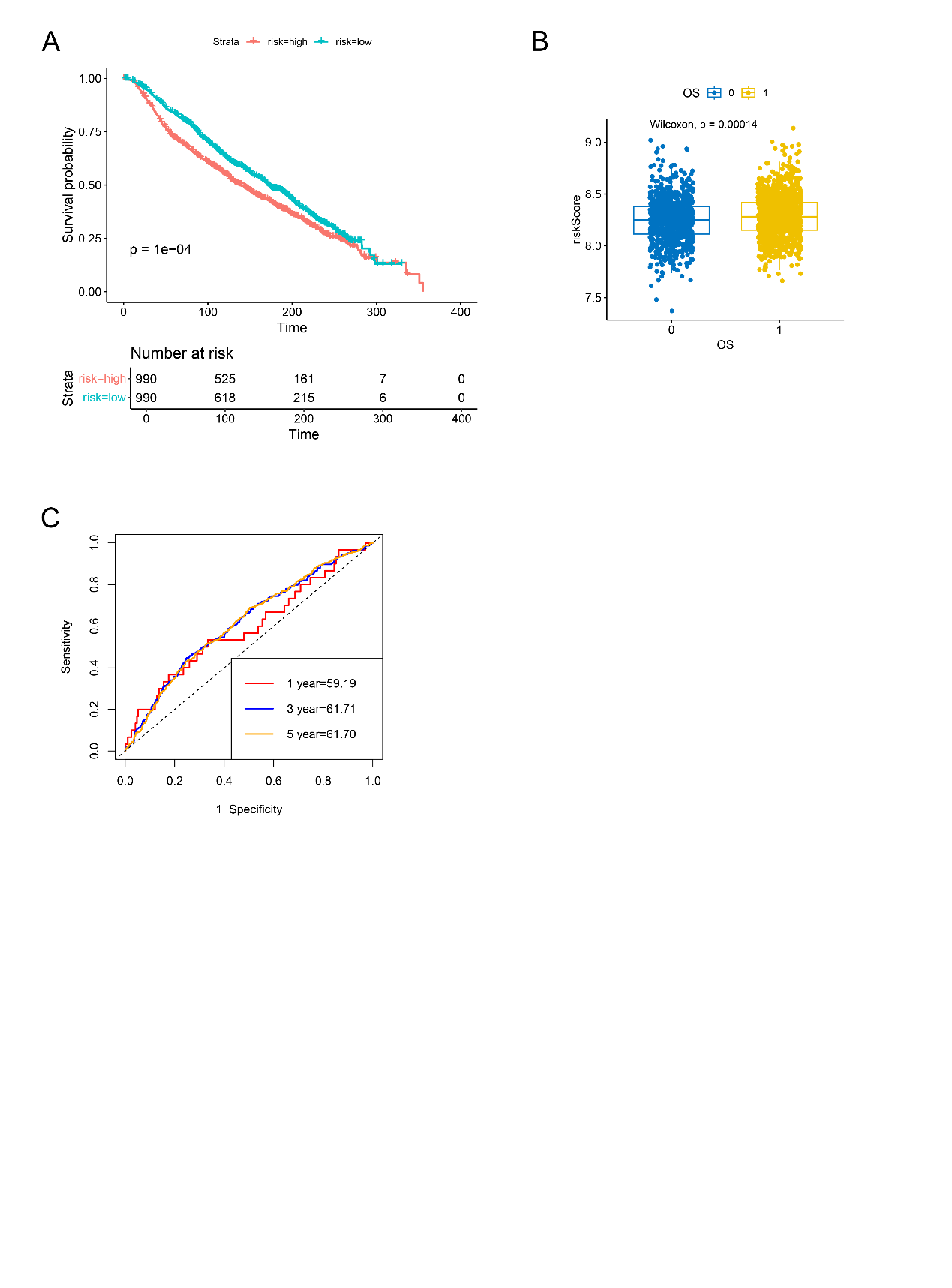


**Supplementary Figures 4.** Circadian rhythm is associated with BRCA overall survival **(A)**Kaplan–Meier curves for patients with high hypoxia and low hypoxia scores. **(B)** Comparison of risk scores in alive and dead patients. **(C)** ROC curves for predicting 1-, 5-, and 10-year OS by risk score.
